# Characterising anthelmintic resistance to benzimidazoles and macrocyclic lactones in gastrointestinal nematodes of dairy cattle

**DOI:** 10.1016/j.ijpddr.2026.100654

**Published:** 2026-06-16

**Authors:** Paul Campbell, Jennifer Mcintyre, Alistair Antonopoulos, Kerry O'Neill, Andrew Forbes, Kathryn Ellis, Roz Laing

**Affiliations:** a School of Biodiversity, One Health and Veterinary Medicine, University of Glasgow, Glasgow, UK; b Kreavet, Kruibeke, Belgium

**Keywords:** Anthelmintic resistance, FECRT, Egg hatch test, *Ostertagia ostertagi*, Amplicon sequencing

## Abstract

Anthelmintic resistance in gastrointestinal nematodes (GIN) of cattle is of increasing concern, but the number of studies monitoring anthelmintic efficacy is insufficient to provide a representative picture. In this study, resistance to macrocyclic lactones (ML) and benzimidazoles (BZ) in GINs from 14 Scottish dairy farms (four non-organic, 10 organic) was evaluated using multiple approaches. The faecal egg count reduction test (FECRT) remains the primary tool for evaluating anthelmintic resistance in the field and was used on the four non-organic farms. Differing methodologies and recent guideline updates complicate the interpretation of FECRT results across studies, as alternative statistical frameworks produce varying confidence intervals that can alter conclusions. Nonetheless, resistance to BZs and MLs in *Ostertagia ostertagi*was consistently detected with all analytical approaches on 3/3 and 4/4 non-organic farms, respectively.

The egg hatch test, combined with nematode differentiation, was used to assess BZ resistance across all 14 farms. The 95% effective concentration values observed at both the population and species levels, especially for *O*. *ostertagi* (EC_95_: 0.045-1.294 μg/ml), were consistent with BZ resistance on 13/14 farms. Mixed amplicon sequencing was applied to 10 populations from seven farms, including both pre- and post-treatment FECRT samples. Resistance-associated polymorphisms in the *β-tubulin isotype-1* gene were detected on 5/7 farms and were present at over 25% frequency in *O. ostertagi*, *Cooperia oncophora*, and *Trichostrongylus* spp. populations.The presence of resistance to both BZs and MLs on 3/4 non-organic farms and higher than expected EC values for BZ on 13/14 farms, showing reduced efficacy of BZ across nearly all farms, including organic, underscoring that anthelmintic resistance is not confined to a single management system. This finding emphasizes the need for sustainable parasite control across all production systems to prevent further development and spread of resistance. Although these data originate from Scotland, the high level of animal movement across the UK suggests that the findings are likely relevant to grazing systems throughout much of the country.

## Highlights

• Resistance of *Ostertagia ostertagi* to benzimidazoles and macrocyclic lactones was detected on most farms.
• Egg hatch tests showed high EC values indicating widespread benzimidazole resistance.
• Amplicon sequencing showed benzimidazole resistance alleles across multiple species.

## Graphical abstract

**Figure:**
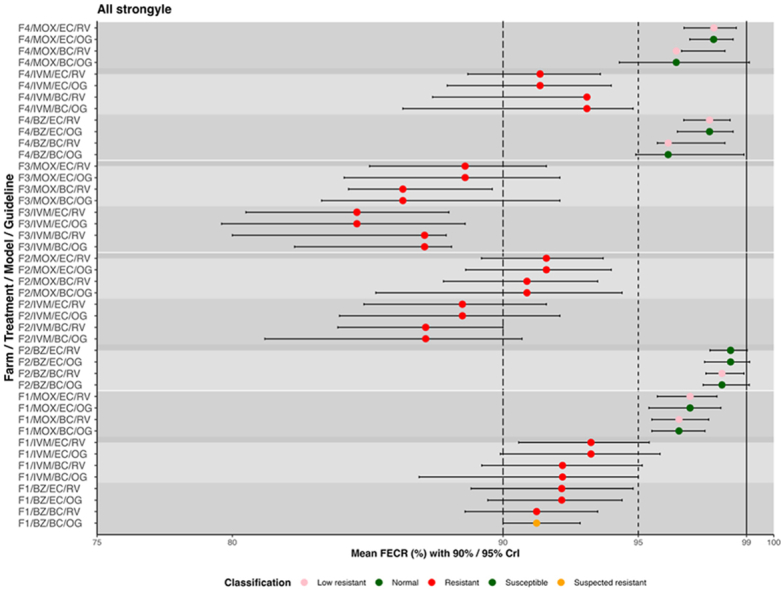

## 1 Introduction

Gastrointestinal nematode (GIN) infections remain a major cause of reduced cattle health, growth, and production efficiency. Although broad-spectrum anthelmintics have underpinned parasite control for over six decades, their extensive use has driven the selection of drug-resistant populations across livestock systems worldwide. Anthelmintic resistance is now widespread in GIN of small ruminants (Rose Vineer et al., 2020), where the rapid expansion of multidrug resistance has been well documented and is increasingly recognised as a threat to cattle production (Bullen et al., 2016; Sauermann et al., 2024; Sutherland and Leathwick, 2011).

In cattle, the lower reported prevalence of resistance in temperate regions may result from typical anthelmintic use practices, which generally allow for relatively high refugia, but may also reflect methodological limitations. Faecal egg counts (FECs) in cattle in temperate regions where *Ostertagia* and *Cooperia* species predominate are typically low and show variable and inconsistent relationships with actual worm burden, consequent to the influence of factors such as host immunity, parasite fecundity and reproductive biology (Sabatini et al., 2023). However, strong correlations between total FEC and adult worm numbers have been demonstrated in some settings, particularly in the sub-tropics, in young cattle infected with specific parasite species, notably *Haemonchus placei*, indicating that the relationship is context-dependent (Teixeira et al., 2021). Consequently, the faecal egg count reduction test (FECRT), the current field standard to characterise anthelmintic susceptibility, has reduced diagnostic sensitivity in cattle (Levecke et al., 2012; Love et al., 2017; Morgan et al., 2022; Playford and Besier, 2025). Despite these challenges, resistance is increasingly reported, and multidrug resistance to benzimidazoles (BZs), macrocyclic lactones (MLs), and levamisole (LEV) has recently been confirmed in *Ostertagia ostertagi* and *Cooperia oncophora* in New Zealand (Sauermann et al., 2024).

Evidence from the UK remains limited. A small number of studies have reported low-to-moderate levels of ML resistance (Bartley et al., 2012; Geurden et al., 2015; McArthur et al., 2011; Stafford and Coles, 1999), whereas BZ resistance appears rare, with only one confirmed case to date (Bartley et al., 2021). However, most surveys involve only a few farms, limiting the ability to generate a representative national assessment. Gastrointestinal nematode infection risk is greatest during the first 2–3 years of life, particularly during the initial period of grazing exposure (often referred to as the first grazing season in seasonal systems) before the development of acquired immunity, after which cattle typically maintain low FECs (Jelinski et al., 2017; O'Shaughnessy et al., 2015). Cattle under two years of age, consequently, shed more eggs and represent the most suitable age group for assessing anthelmintic efficacy using the FECRT. Organic production systems provide an additional perspective, with their restricted use of MLs and emphasis on minimal anthelmintic usage and targeted-selective-treatment strategies (Chylinski et al., 2022; IFOAM, 2014; Takeuchi-Storm et al., 2019). Their GIN populations are expected to have minimal exposure to MLs, with BZ and LEV products as the primary treatments.

To address current knowledge gaps, we investigated the prevalence of anthelmintic resistance in Scottish first-grazing-season (FGS) dairy calves. The FECRT was used to assess the efficacy of commonly administered anthelmintics – ivermectin, moxidectin, and fenbendazole on the non-organic farms, while the egg hatch test was used on both management types. Mixed amplicon sequencing was used on a subset of populations to characterise GIN species composition and quantify the frequency of genetic markers associated with BZ and LEV resistance.

## 2 Methods

### 2.1 Ethics statement

The University of Glasgow MVLS College Ethics Committee (Project No: 200210097) approved all research procedures involving animal use.

### 2.2 Farm selection

All 14 farms were chosen based on specific criteria: located in Scotland; herd size of ≥30 FGS calves; no anthelmintic treatment administered during the current grazing season; and at least two months of grazing before sample collection. Additionally, all farms were required to complete a livestock management survey. The four farms participating in the FECRT were also required to have a cattle crush and handling system available.

All farms were offered free FEC analyses and evaluation of anthelmintic efficacy by FECRT. Farms participating in the FECRT were all non-organic (n = 4), while the egg hatch test was used in all 14 farms, including organic systems (n = 10). The FECRT could not be conducted on the organic farms as no farm routinely treated animals during the grazing season, and treatment solely for conducting a FECRT for this study was not within current regulations. All organic farms participating in this study, were certified to comply with European Organic Regulation (EC 834/2007) and certified by either Scottish Organic Producers Association (SOPA) or Soil Association Certification Ltd.

### 2.3 Farm management survey

A questionnaire was completed during a semi-structured interview, collecting demographic data and information on pasture management and anthelmintic usage. The questionnaire used has been described previously (see Campbell et al., 2025; Supplementary File 1).

### 2.4 Faecal egg count reduction test protocol

All animals for each farm participating in the study were turned out to pasture in May 2023, prior to being housed indoors. From eight weeks post-turnout, FECs were monitored fortnightly until the group mean FEC reached ∼100 eggs per gram (epg). Additionally, bovine lungworm (*Dictyocaulus viviparus*) larvae in faeces and body condition were measured regularly. On the day of treatment (Day 0) on each farm, 15 calves were randomly allocated to a treatment group and received either fenbendazole (FBZ) (Panacur® 10% Oral Suspension; MSD Animal Health) *per os*, subcutaneous IVM (IVOMEC® Classic Injection for Cattle and Sheep; Boehringer Ingelheim), or MOX (Cydectin® 10% LA Solution for Injection; Zoetis) by subcutaneous ear injection, at the manufacturers’ recommended dose rates of 7.5, 0.2, and 1.0 mg/kg body weight respectively. Calves were either weighed individually or their weight estimated using a dairy calf weight band (AHDB), with weights ranging from 187-239 kg. The authors performed dose calculations and administered the anthelmintics accordingly, rounding each dosage to the nearest practical measure based on the formulation: 1 mL for FBZ, 0.1 mL for IVM, and 0.05 mL for MOX.

Faecal samples were collected per rectum from all animals on Day 0 (pre-treatment) and Day 14 (post-treatment). Samples were sealed immediately after collection and transported to the University of Glasgow on the same day. Aliquots of faecal material were stored at 4 °C for FEC analysis, while samples for egg isolation and coproculture were stored at room temperature and processed within 24 h of collection. On all farms, calves from different treatment groups grazed together on the same pastures until Day 14.

### 2.5 Faecal egg count

A faecal egg count was performed on every individual animal sample using a modified salt flotation technique as described in Jackson (1974) with a detection limit and multiplication factor of one epg. Briefly, faecal samples (3 g) were homogenised in 10 mL of water per gram of faeces suspension, passed through a 1 mm sieve, and then rinsed with 5 mL of water to remove debris. The filtrate was then transferred to polyallomer centrifuge tubes and centrifuged at 200 x *g* for 5 min. The supernatant was discarded, and the pellet resuspended in saturated salt solution (SSS) (specific gravity of 1.2), vortexed, and centrifuged at 200 x *g* for 10 min. The surface meniscus was then carefully isolated by clamping the tube below with haemostat clips and transferring the upper layer of the suspension into a cuvette, which was then completely filled with SSS. Cuvettes were read after waiting a minimum of 5 min, and GIN eggs were identified morphologically and counted as either strongyle-type or *Nematodirus* spp.

For each individual animal, the number of strongyle eggs present pre- and post-treatment was used to calculate the percentage reduction in FEC, thus estimating anthelmintic efficacy. In line with the revised FECRT guidelines (Kaplan et al., 2023), a minimum mean of 40 strongyle-type eggs per animal pre-treatment was necessary for a reliable assessment. This threshold was met by conducting one FEC per individual; however, to enable species-specific faecal egg count reduction calculations, it was estimated that three FECs (technical replicates) per sample were required. When the mean FEC of a sample was between 0 and 1 epg, it was always rounded up to 1 epg.

### 2.6 Egg hatch test

Egg hatch tests on all farms participating in the FECRT were conducted using eggs pooled from all pre-treatment animals. For the organic farms, eggs were pooled from freshly voided faeces collected directly from pasture using the methodology described in Campbell et al. (2025).

Eggs were isolated from pooled fresh faeces by sieving, centrifugation, and flotation in SSS. Briefly, 300 g of faeces were mixed with tap water, passed through 500 μm and 210 μm sieves, and centrifuged at 200 x *g* for 5 min. The supernatant was discarded, then a pinch (<0.2 g) of kaolin was added to the pellet, and the mixture was vortexed before being resuspended in SSS. After centrifugation at 200 x *g* for 10 min, the polyallomer tubes were clamped to isolate the eggs, which were then collected in a 38 μm sieve, rinsed thoroughly with deionised water, and examined microscopically to confirm that embryonation had not yet begun.

Each sample was tested in triplicate using six concentrations of thiabendazole (TBZ) dissolved in 0.5% DMSO (0.01, 0.025, 0.05, 0.1, 0.25 and 0.5 μg TBZ/mL) and a negative control (no drug, 0.5% DMSO), also in triplicate. The EHT was performed following the protocol described by Samson-Himmelstjerna et al. (2009). After 48 h, the test was terminated with Lugol's iodine stain, the contents of each well were transferred to a Petri dish, and all eggs and larvae were counted. The contents from each well were then pooled by TBZ concentration for subsequent species identification. Up to 94 eggs and 94 larvae were identified to species level for each TBZ concentration using a multiplex PCR method (Bisset et al., 2014), described in 2.7.

### 2.7 Coproculture and species identification by PCR

Pooled larval coprocultures from each pre- and post-treatment group were prepared by hand-mixing approximately 300 g of faeces with vermiculite to produce a well-aerated, uniform, paste-like consistency. The coprocultures were incubated at 27 °C for 14 days and sprayed with water to maintain humidity for L_3_ development. After incubation, larvae were harvested using a modified Baermann technique as described in Roberts and O'Sullivan (1950); pooled aliquots of L_3_ in water were stored at −80 °C.

Crude lysates were prepared from single strongyle eggs or larvae in 96-well plates using a modified proteinase K lysis reaction for individual strongyles identified by PCR, as described by Campbell et al. (2025). The crude lysates were then diluted 1:20 with nuclease-free water.

For each pooled sample, a minimum of 94 recovered larvae/eggs were identified to species level by PCR targeting the ITS-2 region. A multiplex PCR method (Bisset et al., 2014) was employed, using primers designed to amplify the strongyle ITS-2 region and species-specific regions for GIN species of interest (see Supplementary File 1). The reaction set included primers targeting*, O. ostertagi, C. oncophora, Trichostrongylus axei, Oesophagostomum venulosum,* and *Haemonchus spp.*. The multiplex PCRs were performed as described by Campbell et al. (2025), in 96-well plates with 94 individual worm lysates, one DNA-negative control (no gDNA template), and one lysis-negative control (lysate without larva).

### 2.8 Genomic DNA isolation and amplicon sequencing library preparation

Pooled populations of GINs for amplicon sequencing were selected based on the availability of sufficient larvae, anthelmintic efficacy (FECRT), and TBZ sensitivity (EHT), to provide data representative of the range management systems and anthelmintic susceptibility.

Genomic DNA from pools of ∼3000 L_3_ was isolated using the Monarch® Spin gDNA Extraction Kit (T3010S) following the manufacturer's instructions, with a final elution in 30 μl of buffer (10 mM Tris-HCl, pH 9.0, 0.1 mM EDTA). The isolated gDNA was normalised to a concentration of 25 ng/μL, and all samples were stored at 4 °C.

Mixed-amplicon sequencing libraries were generated through individual PCR amplification of the following loci: ITS-2, *β-tubulin isotype-1*, *β-tubulin isotype-2*, and *acr-8*, with the latter two being specific to *O. ostertagi* only. The latter three loci have been associated with anthelmintic resistance phenotype for BZ (*β-tubulin* loci) or LEV (*acr-8*). No *O. ostertagi* genome assembly was available at the time of this study. Therefore, to identify candidate loci for amplification and NGS analysis, a library of full-length *O. ostertagi* transcripts generated from isoform sequencing (Iso-Seq) was employed (PRJNA898386 (Price et al., 2024)). The phylogenetic orthology inference tool OrthoFinder v2.5.4 (Emms and Kelly, 2015) was used to identify candidate loci based on orthology, using transcripts from closely related nematodes: *H. contortus* (PRJEB506, MHCO3ISE_4.0 (Doyle et al., 2020)), *Teladorsagia circumcincta* (PRJNA72569, T_circumcincta.14.0 (Coghlan et al., 2019)), *C*. *oncophora* (PRJEB196 (Abubucker et al., 2014)), and *Caenorhabditis elegans* (PRJNA13758, WBCEL235 (Harris et al., 2019)). Candidate *O. ostertagi* sequences were aligned to *H. contortus*, *T. circumcincta*, and *C. elegans* sequences for the respective candidate genes using Geneious v2023.1 and ranked by their homology. Exon junctions were predicted through comparative analysis among these three species. Primers were designed to lie entirely within predicted exons and to ensure the expected amplicon size did not exceed 250-300 bp. For a complete list of NGS primers used in this study, see Supplementary File 2.

Amplification of ITS2 and *β-tubulin isotype-1* loci included pan-nematode primer pairs developed by Avramenko et al. (2019, 2015). The PCR reactions (50 μL total volume) contained: 10 μL 5× Phusion GC buffer (New England Biolabs), 1 μL of 10 mM dNTPs, 2.5 μL of 10 μM of each forward and reverse primer (or primer mix), 0.5 μL of Phusion DNA Polymerase, 1 μL of gDNA, and 32.5 μL of nuclease-free water. All PCR steps were performed following best practices to minimise aerosol formation, including the use of filter pipette tips, working in a PCR cabinet, and sealing PCR plates with adhesive seals.

The unindexed amplicon libraries were submitted to the University of Glasgow MVLS SRF for Illumina MiSeq amplicon sequencing. Stage-2 indexing PCRs, library quantification, normalisation, pooling, and denaturing were performed according to Illumina's 16S Metagenomic Sequencing Library Preparation protocol (Illumina Inc., USA). Illumina 250 bp paired-end (250 PE) sequencing was performed on an Illumina MiSeq platform using MiSeq Reagent Kits v2.0 (500 cycles). The MiSeq was configured to generate only FASTQ files with no post-run analysis. Samples were automatically demultiplexed by the MiSeq using the provided index combinations.

### 2.9 Bioinformatic analysis

All loci were analysed separately. Briefly, amplicon sequence variants (ASVs) were identified for each locus using *dada2* v1.30.0 (Callahan et al., 2016) in RStudio. Filtering was employed to remove reads containing unresolved nucleotides as well as reads exceeding the expected error number and size range: filterAndTrim (run parameters: maxN = 0, maxEE = c(2,2), truncQ = 2, minLen = 50, rm.phix = TRUE, matchIDs = TRUE). This filtered dataset was then used for error training, learnErrors(), and error correction (denoising). The paired reads were merged, and chimeric sequences were identified before removal using removeBimeraDenovo(). A final table was produced for all the ASVs identified, along with their frequencies within the dataset. The ITS-2 datasets were analysed using the Nemabiome workflow and correction factors for species-specific nematodes (Avramenko et al., 2019;Avramenko et al., 2015)

### 2.10 Statistical analysis

For all FEC datasets, statistical analysis was performed in R Statistical Software (v4.5.0; R Core Team, 2021) to calculate the faecal egg count reduction (FECR) using the R packages *eggCounts* (v2.5-1; Torgerson et al., 2014) to estimate the FECR with 90% and 95% credible intervals (CrI), and *bayescount* (v0.9.99-9; Denwood et al., 2023) to calculate the 90% CrI. The output of the *eggCounts* and *bayescount* packages were interpreted based on the original FECRT guidelines described by Coles et al. (1992) and the revised guidelines (RV) described by Kaplan et al. (2023). The two guidelines are summarised in Table 1. To quantitatively compare the agreement between statistical models and the original and revised FECRT guidelines, weighted Cohen's κ coefficients were calculated using *kappa2(weights = "quadratic")* (Normal < Inconclusive < Low resistant < Resistant) and interpreted according to McHugh (2012).

**Table 1:** Comparison of the classification criteria for the faecal egg count reduction test.

Interpretation	Lower 95% FECR CL	FECR estimate (%)	Upper 95% FECR CL
Original guidelines described by Coles et al. (1992)			
**Normal**	>90	>95	NR
**Suspected susceptible**	<90	>95	NR
**Suspected resistant**	>90	<95	NR
**Resistant**	<90	<95	NR

Interpretation	Lower 90% FECR CrI	FECR estimate	Upper 90% FECR CrI
Revised guidelines described by Kaplan et al., 2023			
**Susceptible**	>95	NR	>99
**Inconclusive**	<95	NR	>99
**Low resistant**	>95	NR	<99
**Resistant**	<95	NR	<99

FECR; faecal egg count reduction, CL; confidence limit, CrI; credible interval, NR; not relevant.

The EC_50_ (effective concentration for 50% inhibition) and EC_95_ values for each EHT were calculated using the R package *drc* (v3.0-1; Ritz et al., 2015) and the *drm()* function using the LL.4 model. The *EDcomp()* function compares effective doses derived from dose-response curves and reports p-values reflecting the statistical significance of the differences between groups (p ≤ 0.05 considered significant). Farm 3, which had equivalent EC values to those of sensitive isolates reported by Samson-Himmelstjerna et al. (2009), was designated as a 'susceptible isolate'. Subsequently, resistance ratios (RR) were calculated as the EC value of each sample divided by the EC value of Farm 3.

## 3 Results

### 3.1 Farms included in the study and their parasite management

The basic farm demographics and history of anthelmintic use are summarised in Table 2. Fourteen farms participated in this study: four farms took part in the FECRT, and all farms participated in the egg hatch test. All farms operated as commercial dairy farms, with most also engaged in dairy-beef production (13/14), and half (7/14) also kept sheep. The groups of FGS calves ranged from 29-62 individuals, all of which were spring-born and aged between four and 7 months at the time of sampling, with 71%-100% being female. All non-organic farms reported using ML anthelmintics exclusively during the previous seven years, while organic farms exclusively used BZ products, and no farms reported using levamisole. The 10 organic farms sampled in the study account for approximately 48% (10/21) of Scotland's organic dairy farms (Agriculture and Rural Economy Directorate Scotland, 2025). All farms participated in administering anthelmintics at the group level. Non-organic farms reported an average of two treatments annually, mainly one mid-grazing season treatment while at pasture and one at housing at the end of the grazing season. Among organic farms, 6/10 averaged one group treatment per year, while four reported fewer than one treatment annually. The farms used three main treatment regimens, which we define as follows:

- Neo-suppressive: treatment to limit the establishment of a parasitic infection and minimise pasture larval contamination
- Prophylactic: treatment of an at-risk group in anticipation of clinical or production-limiting parasitism based on previous management experience, but without the use of diagnostic indicators
- Test-and-treat: treatment based on a FEC result that may be production-limiting

**Table 2:** Farm demographics.

Farm	Organic status	Dairy system	Calving pattern	No. of calves in study group	Anthelmintic compounds used in previous 7 years	Average number of anthelmintic treatments (group) per year	Treatment strategies previously employed
1	Non-organic	Dairy & beef	Dual-block	46	IVM, DOR	2	PT
2	Non-organic	Dairy & beef	Dual-block	48	IVM	2	PT,
3	Non-organic	Dairy	AYR	33	MOX LA, IVM	2	PT, NS
4	Non-organic	Dairy, beef, & sheep	Spring-block	45	MOX LA, MOX, IVM	2	PT, NS
5	Organic	Dairy & beef	AYR	37	FBZ	1	TT
6	Organic	Dairy, beef, & sheep	Dual-block	44	FBZ	1	TT, PT
7	Organic	Dairy & beef	AYR	29	FBZ, ABZ	<1	TT
8	Organic	Dairy, beef, & sheep	AYR	33	FBZ	1	TT, PT
9	Organic	Dairy, beef, & sheep	Dual-block	38	FBZ	1	TT,
10	Organic	Dairy & beef	Dual-block	59	FBZ	<1	TT
11	Organic	Dairy, beef, & sheep	AYR	39	FBZ, ABZ	1	TT
12	Organic	Dairy & beef	AYR	40	FBZ	<1	TT, PT
13	Organic	Dairy, beef, & sheep	Dual-block	39	FBZ	1	TT, PT
14	Organic	Dairy, beef, & sheep	Dual-block	46	FBZ	<1	TT

FECRT; Faecal egg count reduction test, IVM; Ivermectin, DOR; Doramectin, ST; Strategic treatment, AYR; All-year-round, MOX LA; Moxidectin long-acting, PT; Prophylactic treatment, MOX; Moxidectin, FBZ; Fenbendazole, TT; Test and Treat Dual block: spring and autumn calving.

All non-organic farms employed some form of prophylactic treatment regimen, two out of four conventional farms (Farms 3 and 4) also employed a neo-suppressive control strategy by administering a long-acting moxidectin (MOX) injectable. All organic farms adopted a test-and-treat approach based on faecal egg counts (FECs), while four out of ten organic farms additionally employed a prophylactic treatment strategy, administering treatments in anticipation of a significant parasite challenge and/or burden. Effective quarantine procedures were not routinely practised on any farm in this study: five farms reported administering quarantine treatments to newly purchased cattle, but all indicated that they did not routinely treat every animal introduced to the herd, with no post-treatment FEC.

### 3.2 Non-modelled data

**Fig. 1:**
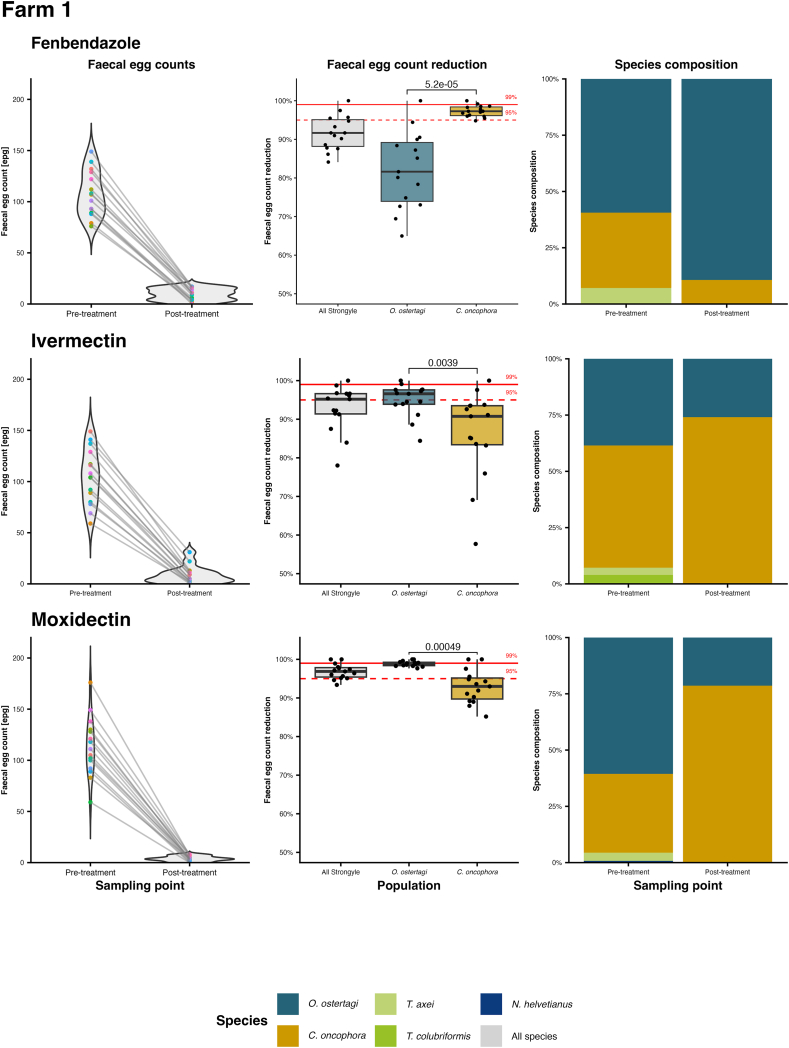
**Farm 1: paired faecal egg count reduction, anthelmintic efficacy and relative species abundance of gastrointestinal nematode communities, pre- and post-treatment on Farm 1.** Species identity was assigned by ITS-2 rDNA multiplex PCR of a pool of L_3_ harvested from coprocultures of each cohort and time point. A minimum of 94 L_3_ were identified per pooled coproculture. The violin plots with paired points represent the probability and distribution of strongyle-type faecal egg counts (FECs) pre- and post-treatment. Faecal egg counts were conducted using a modified salt flotation technique with a sensitivity of 1 epg. The boxplots represent the faecal egg count reduction estimates for each individual, based on the FEC and interpolated species compositions.

**Fig. 2:**
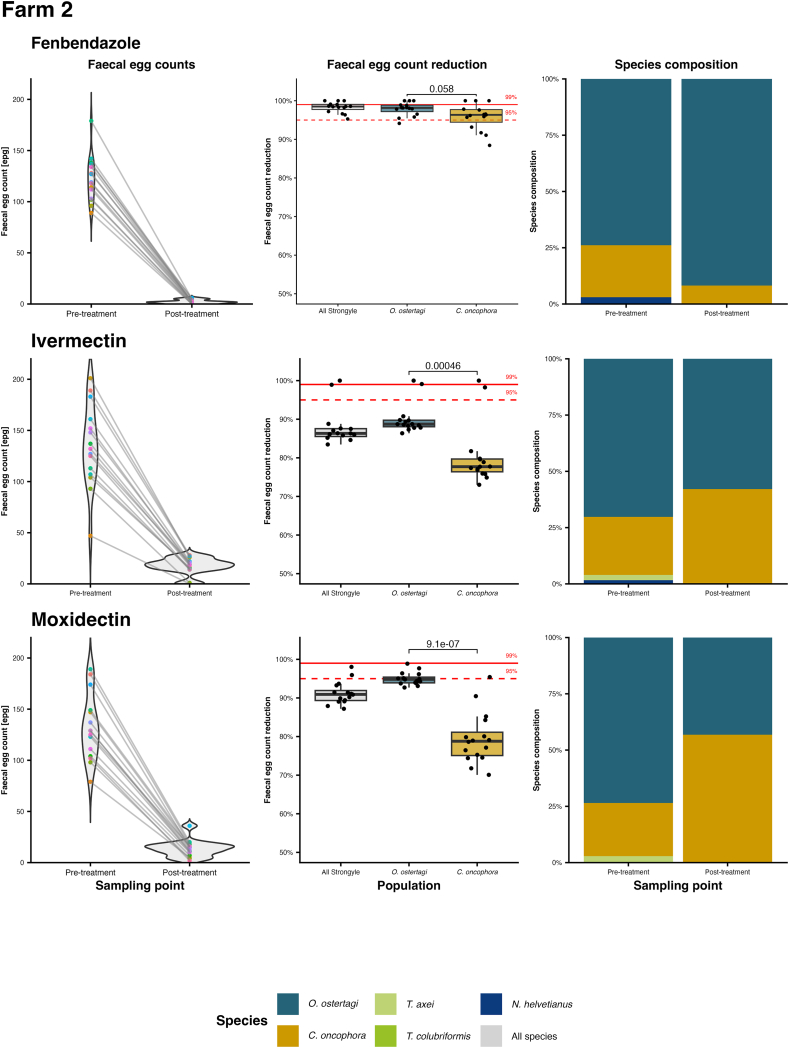
**Farm 2: paired faecal egg count reduction, anthelmintic efficacy and relative species abundance of gastrointestinal nematode communities, pre- and post-treatment on Farm 2.** Species identity was assigned by ITS-2 rDNA multiplex PCR of a pool of L_3_ harvested from coprocultures of each cohort and time point. A minimum of 94 L_3_ were identified per pooled coproculture. The violin plots with paired points represent the probability and distribution of strongyle-type faecal egg counts (FECs) pre- and post-treatment. Faecal egg counts were conducted using a modified salt flotation technique with a sensitivity of one epg. The boxplots represent the faecal egg count reduction estimates for each individual, based on the FEC and interpolated species compositions.

**Fig. 3:**
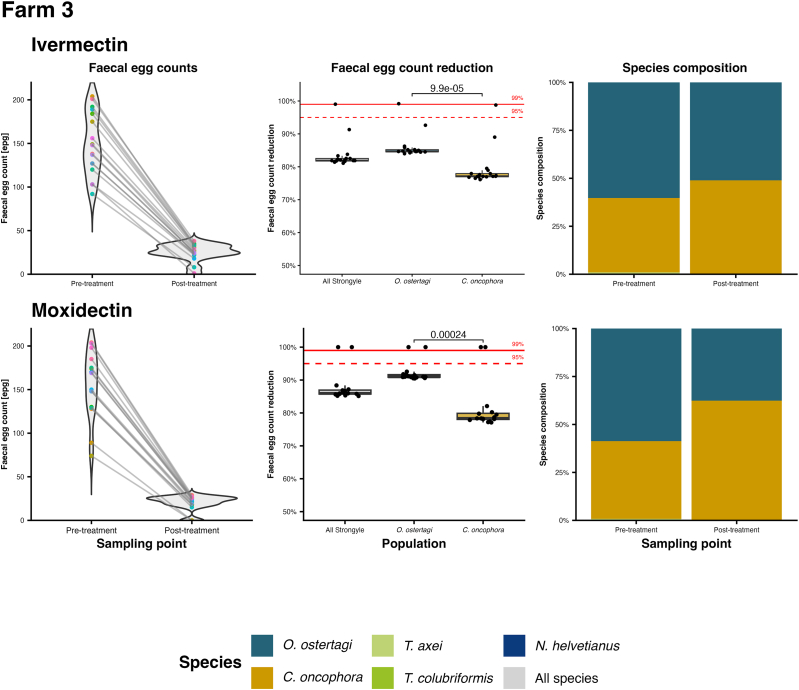
**Farm 3: paired faecal egg count reduction, anthelmintic efficacy and relative species abundance of gastrointestinal nematode communities, pre- and post-treatment on Farm 3.** Species identity was assigned by ITS-2 rDNA multiplex PCR of a pool of L_3_ harvested from coprocultures of each cohort and time point. A minimum of 94 L_3_ were identified per pooled coproculture. The violin plots with paired points represent the probability and distribution of strongyle-type faecal egg counts (FECs) pre- and post-treatment. Faecal egg counts were conducted using a modified salt flotation technique with a sensitivity of 1 epg. The boxplots represent the faecal egg count reduction estimates for each individual, based on the FEC and interpolated species compositions.

**Fig. 4:**
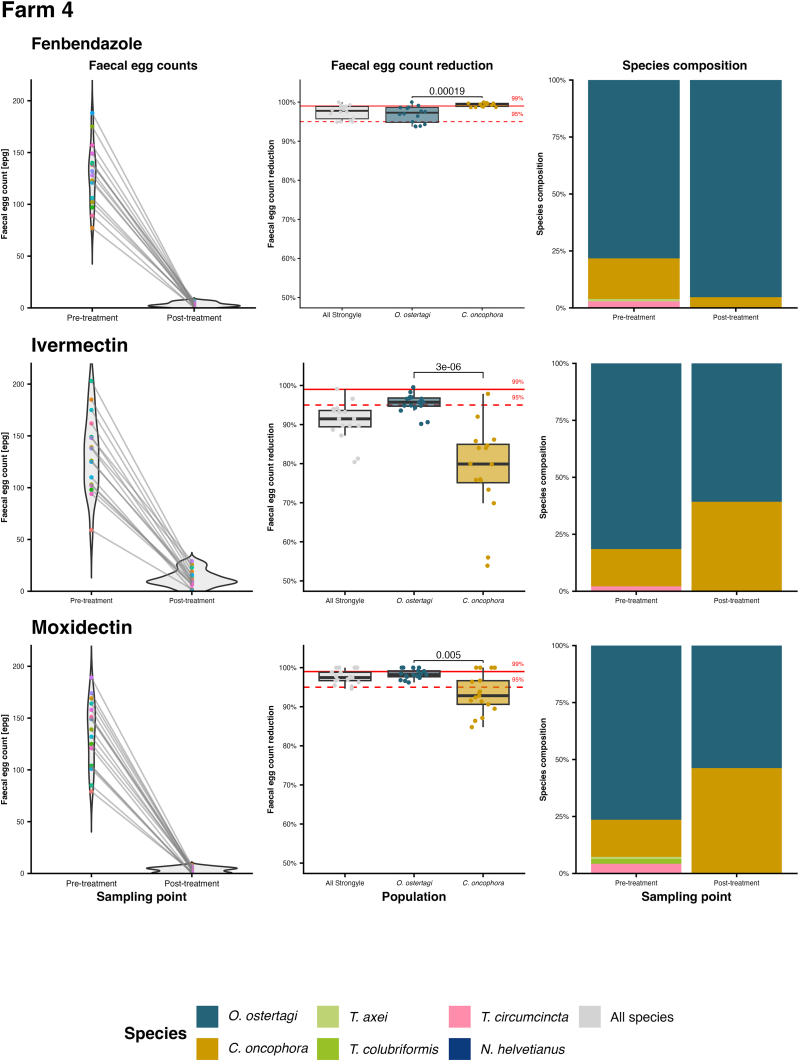
**Farm 4: paired faecal egg count reduction, anthelmintic efficacy and relative species abundance of gastrointestinal nematode communities, pre- and post-treatment on Farm 4.** Species identity was assigned by ITS-2 rDNA multiplex PCR of a pool of L_3_ harvested from coprocultures of each cohort and time point. A minimum of 94 L_3_ were identified per pooled coproculture. The violin plots with paired points represent the probability and distribution of the strongyle-type faecal egg counts (FECs) pre- and post-treatment. Faecal egg counts were conducted using a modified salt flotation technique with a sensitivity of one epg. The boxplots represent the faecal egg count reduction estimates for each individual, based on the FEC and interpolated species compositions.

On all four FECRT farms, FECs were reduced across all populations following treatment with any anthelmintic drug (Fig. 1, Fig. 2, Fig. 3, Fig. 4). Violin plots illustrating paired pre- and post-treatment FECs are presented in the first column of Fig. 1, Fig. 2, Fig. 3, Fig. 4. As expected, pre-treatment data for all treatments were over-dispersed, with substantial variance in FEC. The paired FECR for each individual is shown in the second column; notably, a difference in FECR between *O. ostertagi* and *C. oncophora* was observed across treatments.

Species composition of each strongyle population was determined by multiplex PCR, revealing six species across five genera. Post-treatment species proportion patterns differed by anthelmintic class. Following fenbendazole (FBZ) treatment, the proportion of *O. ostertagi* consistently increased, whereas after MOX treatment, the proportion of *C. oncophora* consistently increased. Following ML treatment, *C. oncophora* proportion increased in six of the eight populations, while *O. ostertagi* proportion increased in the Farm 1 and Farm 4 populations.

In all pre-treatment populations, *O. ostertagi* was the most prevalent species, with *C. oncophora* consistently the second most prevalent. In all post-treatment populations, only two species were observed: *O. ostertagi* and *C. oncophora*.

### 3.3 Comparison and interpretation of the faecal egg count reduction test between statistical methods and guidelines

The interpretation of the FECRT using the previous WAAVP guidelines for detecting anthelmintic resistance (Coles et al., 1992) was compared with the recently published revised guidelines (Kaplan et al., 2023). For this purpose, the categories for determining anthelmintic efficacy from the original guidelines (OG) - i.e., reduced, suspected resistant, suspected susceptible, and normal - were considered equivalent to those of resistant, low resistant, inconclusive, and susceptible, respectively, from the current revised guidelines. For all comparisons of guidelines and FECRT modelling packages, see Supplementary File 3.

**Fig. 5:**
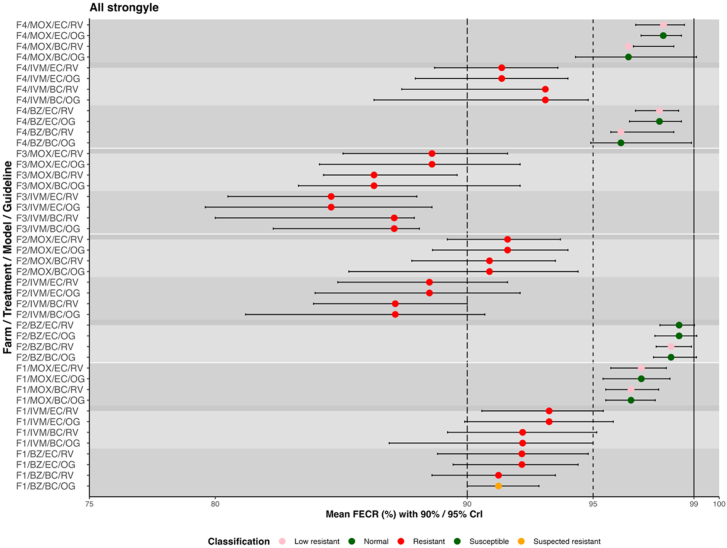
**Comparison of the *eggCounts* and *bayescount* faecal egg count reduction estimates for the entire strongyle population.** Faecal egg count reductions (FECR) with credible intervals (CrIs) for anthelmintic treatment against the entire strongyle population on Farms 1-4 (F1-F4). The CrIs were calculated using either *eggCounts* (EC) or *bayescount* (BC) models and interpreted according to either the revised guidelines (R) for the faecal egg count reduction test (Kaplan et al., 2023) with 90% CrIs, or the original guidelines (OG) (Coles et al., 1992) with 95% CrIs. Each point represents the mean FECR, with colour indicating the resistance status for the entire strongyle population: green, susceptible/normal; red, resistant; pink, low resistant/suspected resistant; orange, inconclusive/suspected susceptible.

When assigning a resistance status to the entire strongyle population against FBZ using the revised guidelines, only one scenario resulted in a susceptible classification (F2/BZ/EC/RV), as shown in Fig. 5. Consistency across all FBZ scenarios was observed only in the status assigned to Farm 1, which was classified as resistant regardless of the model or guidelines used. In all IVM treatment scenarios across all farms, resistance was determined to be present within the strongyle population. The MOX-treated populations of Farms 2 and 3 were consistently classified as resistant in all scenarios, whereas the MOX-treated populations of Farms 1 and 4 were classified as 'low resistant' under the revised guidelines and 'normal' under the original guidelines.

Based on the revised guidelines both statistical methods agreed for 10/11 FECRTs (Table 3), with the BZ FECRT on Farm 3 estimated to be susceptible by *eggCounts* and as low resistance by *bayescount*. This resulted in a Cohen's κ value of 0.656, indicating substantial agreement.

**Table 3:** The inter-rater agreement between *eggCounts* and *bayescount* results based on the revised guidelines for the faecal egg count reduction test (Kaplan et al., 2023) for the entire strongyle population.

bayescount	eggCounts			
	Susceptible	Inconclusive	Low resistant	Resistant
**Susceptible**	-	-	-	-
**Inconclusive**	-	-	-	-
**Low resistant**	1	-	3	-
**Resistant**	-	-	-	7
	Cohen's k = 0.656 (substantial agreement)			

Comparing interpretations of the FECRT results between the original and revised guidelines was conducted using only the *eggCounts* data. The interpretation of results using both guidelines agreed for 8/11 datasets (Table 4). Three datasets classified as low resistance by the revised guidelines were classified as normal (susceptible) by the original guidelines. This produced the same level of agreement as between both modelling packages and the revised guidelines.

**Table 4:** Inter-rater agreement between the original guidelines (Coles et al., 1992) and the revised guidelines (Kaplan et al., 2023) based on the faecal egg count reduction test for the entire strongyle population analysed using *eggCounts*.

Original guidelines	Revised guidelines			
	Susceptible	Inconclusive	Low resistance	Resistant
Normal	1	-	3	-
Suspected susceptibility	-	-	-	-
Suspected resistance	-	-	-	-
Resistance	-	-	-	7
	Cohen's k = 0.656 (substantial agreement)			

When comparing inter-rater agreement between the statistical models using the revised guidelines' interpretations of the FECRT for the interpolated *O. ostertagi* and *C. oncophora* populations, the *O. ostertagi* populations were classified as having the same status as the entire strongyle populations across all treatments and models (Supplementary File 4). This yielded a Cohen's k of 0.656, indicating substantial agreement between both statistical models for this species. In contrast, *C. oncophora* populations were always classified as resistant or low resistant, and the inter-rater agreement for the *C. oncophora* populations was perfect, with a Cohen's k value of 1, consistently assigning ten populations as resistant and one as low resistant.

Comparing the inter-rater agreement between the original and revised guidelines' interpretations using the *eggCounts* model of the interpolated species datasets, there was fair agreement between the guidelines when assigning the resistance status to *O. ostertagi* populations, with a Cohen's k value of 0.333. Three populations were initially classified as normal by the original guidelines, but were classified as low-resistant by the revised guidelines, and one population that was initially classified as normal was also classified as resistant. The inter-rater agreement for the *C. oncophora* populations was lower, with a Cohen's k value of 0.19, consistently assigning resistance to eight populations; however, it also classified two resistant populations as normal by the original guidelines, as well as a low-resistant population and a normal population, indicating only slight agreement between the guidelines.

### 3.4 Egg hatch test dose-response

**Fig. 6:**
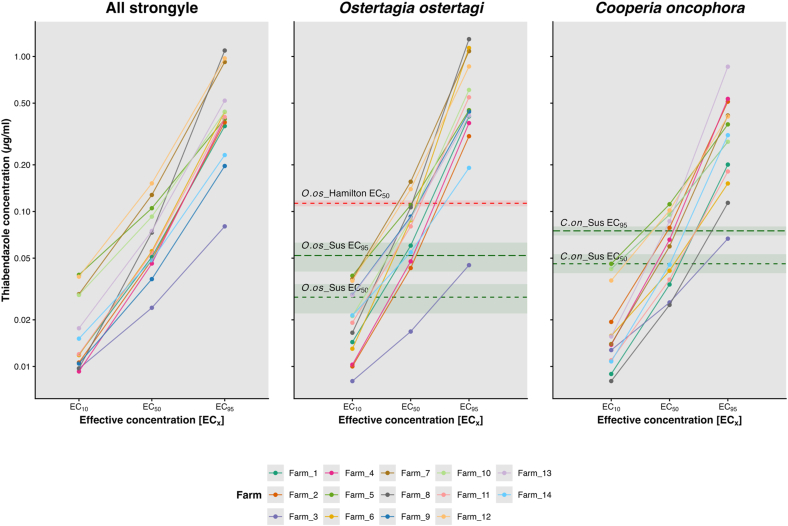
Nematode effective concentration estimates (μg/mL thiabendazole) for each strongyle and interpolated species population. The effective concentrations [EC] were estimated from the respective LL.4 dose-response curve model. The green dashed line represents the reported EC_50_ and EC_95_ of a fully susceptible *Ostertagia ostertagi* and *Cooperia oncophora* isolate, with the standard error of the mean (Demeler et al., 2013). The red line represents the reported EC_50_ of a resistant *O. ostertagi* population ± SEM.

Sufficient numbers of eggs were collected from all organic farms and pre-treatment FECRT populations to perform the egg hatch test. The mean number of eggs added to each well was 207, and the mean hatch proportion in the control wells was 92.6%. The dose-response curves for all populations (all strongyles, *O. ostertagi*, and *C. oncophora*) are shown in Supplementary File 5, with the effective concentrations displayed in Fig. 6. The interpolated effective concentrations for *O. ostertagi* were 0.017–0.157 μg/mL (EC_50_) and 0.045–1.293 μg/mL (EC_95_), and for *C. oncophora*, 0.025–0.111 μg/mL (EC_50_) and 0.416–0.859 μg/mL (EC_95_). The relative RR was also calculated for each species, with Farm 3 used as the sensitive isolate. The RR ranged from 4.24-28.71 and 1.70-12.87 for *O. ostertagi* and *C. oncophora*, respectively. For the specific EC_50_, EC_95_, and RR of each population, see Supplementary File 6.

### 3.5 Amplicon sequencing

Sufficient numbers of larvae were obtained from all treatment groups pre-treatment (range 20,000-83,000) and post-treatment (range 2,300-5,100) to conduct mixed amplicon sequencing of all loci. Ten populations were subjected to the mixed amplicon sequencing marker panel based on larval abundance, parasite management practices, and the likelihood of resistance, as determined by either FECRT or EHT. Four GIN populations from organic dairy farms and six paired pre- and post-treatment FECRT sample populations (FBZ, IVM, MOX) were selected.

#### 3.5.1 Nemabiome

**Fig. 7:**
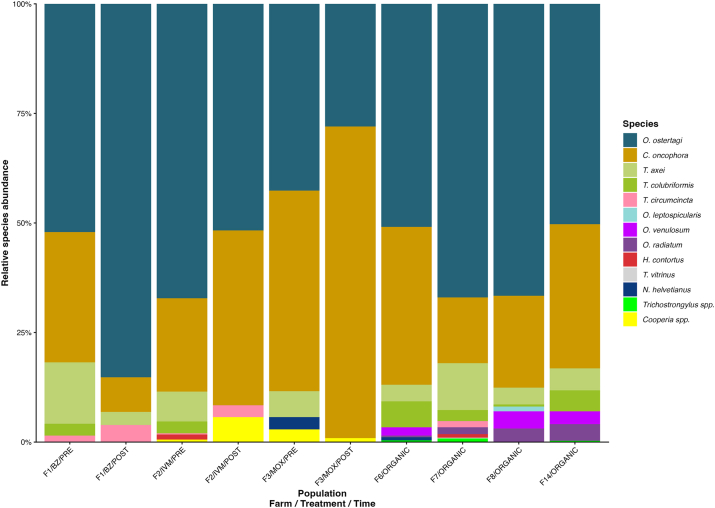
**Gastrointestinal nematode composition determined by ITS-2 nemabiome metabarcoding.** Relative species abundance of gastrointestinal nematode communities, determined by ITS-2 rDNA nemabiome metabarcoding, of pools of L_3_ collected from individual coprocultures of pre- and post-faecal egg count reduction test populations and free-catch pasture samples from organic farms. A minority of ASVs could only be identified to the genus level: *Cooperia* spp. and *Trichostrongylus* spp. groups.

We used ITS-2 nemabiome metabarcoding to determine the species composition of the GIN populations and identified 105 amplicon sequence variants (ASVs), classified into eleven taxa at either the genus (3.7%) or species level (96.3%). A minority of ASVs (n = 5) could not be classified to the species level but were identified as belonging to the genera *Cooperia* (n = 3) and *Trichostrongylus* (n = 2). The most prevalent species identified were *O. ostertagi* and *C. oncophora*, observed in every population (see Fig. 7). *Trichostrongylus axei* was observed on every farm but was absent in both the post-IVM and post-MOX sample populations, while *T. colubriformis* was observed on six farms. *Oesophagostomum* spp. were only observed in GIN populations from organic farms (n = 4), and *Haemonchus contortus* was observed on two farms. In terms of relative abundance, *O. ostertagi* was the most abundant species in the majority of sample populations (8/10) and the most abundant species on all farms except Farm 3. Comparing changes in species abundance pre- and post-BZ treatment, *O. ostertagi* and *Teladorsagia circumcincta* increased from 53.1%-85.2% and from 1.5%-3.9%, respectively, while *C. oncophora* decreased from 29.7%-7.9%. Comparing the effect of ML products, *O. ostertagi* decreased from 67.2%-51.7% and from 42.6%-28% post-IVM and MOX treatment, respectively, while *C. oncophora* increased from 21.3%-39.9% and from 45.8%-71.1%, respectively.

#### 3.5.2 Frequency of *β-tubulin isotype-1* resistance-associated polymorphisms

**Fig. 8:**
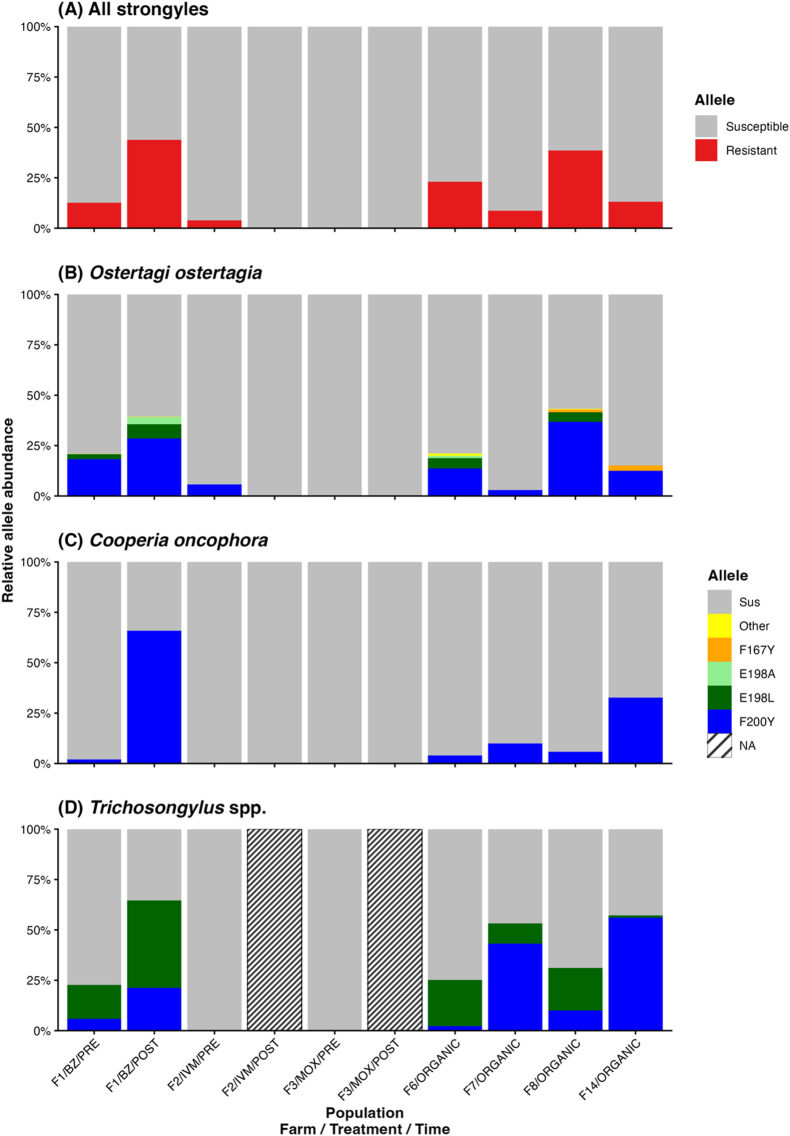
**Frequency and prevalence of *β-tubulin isotype-1* gene resistance alleles.** The relative proportions of the *β-tubulin isotype-1* gene resistance allele frequencies across 10 populations from seven different farms for all strongyles and three nematode species. (A) All strongyles; (B) *Ostertagia ostertagi*; (C) *Cooperia oncophora*; (D) *Trichostrongylus* spp. Susceptible alleles (F167, E198 and F200) are displayed in grey, while previously described resistance-associated polymorphisms (F167Y (TTC > TAC), E198A (GAA > GCA), E198L (GAA > TTA) and F200Y (TTC > TAC)) are marked in red for all strongyles, and in orange, green, light green, and blue respectively, indicating the resistance allele frequency per species. All other identified non-synonymous polymorphisms were grouped and displayed in yellow. Absence of alleles is represented by a diagonal stripe (NA).

Between 4385 and 6211 (mean = 5381) sequence reads for the *β-tubulin isotype-1* loci were generated from each GIN sample population using the mixed amplicon sequencing panel and screened for BZ-resistance polymorphisms at codons 167, 198, and 200. Sequences mapped to *O. ostertagi*, *T. axei*, *T. colubriformis*, *T. vitrinus*, *C. oncophora*, *T. circumcincta*, *H. contortus*, *C. curticei*, *O. leptospicularis*, and *Oesophagostomum* spp. *Ostertagia ostertagi* and *C. oncophor*a *β-tubulin isotype-1* were identified in all samples (Fig. 8). Benzimidazole resistance alleles were present to some degree in all strongyle populations from organic farms and were detected in seven of the ten sequenced sample populations. The majority of *O. ostertagi* resistance alleles detected were F200Y (TTC > TAC), as well as two resistance alleles at codon 198, E198A (GAA > GCA) and E198L (GAA > TTA). However, the F167Y (TTC > TAC) polymorphism was also present in organic Farms 8 and 14 at very low frequencies of 1.3% and 2.6% relative read abundance, respectively. All *C. oncophora* resistance alleles were the F200Y variant, present at a mean frequency of 11.2% relative read abundance, but showed high variability across farms, ranging from 2%-65.8%. No non-synonymous polymorphisms were detected at codons 167 or 198. *Trichostrongylus* spp. (*T. axei*, *T. colubriformis*, *T. vitrinus*) alleles were detected on all farms but were absent in the post-ML treatment populations. The F200Y polymorphism was detected at a mean frequency of 13.9% in *Trichostrongylus* spp., while the E198L polymorphism was present at a highly variable frequency, with a mean of 11.5%, ranging from 1.1%-43.4%. Comparing the effect of FBZ treatment on allele frequency, the total frequency of resistance alleles for all strongyles increased from 12.7%-43.8%, while for *O. ostertagi*, *C. oncophora*, and *Trichostrongylus* spp., the frequencies increased from 20.8%-35.6%, 2%-65.8%, and 22.6%-64.6%, respectively.

No clear association could be observed between BZ-resistance allele relative read abundance and EC values in either *O. ostertagi* or *C. oncophora* (Supplementary File 7). When the EC_50_ and EC_95_ estimates from the pre-treatment populations were compared with the corresponding resistance-allele relative read abundancies, no linear or second-order model was able to fit the data, R^2^ < 0.319.

#### 3.5.3 Frequency of *β-tubulin isotype-2* and *acr-8* resistance-associated polymorphisms

A second BZ phenotype associated gene, *β-tubulin isotype-2*, was screened for polymorphisms at codons 198 and 200; all ASVs generated were unambiguous for *O. ostertagi*, and no resistance-conferring alleles were observed at the codon. Unambiguous *O. ostertagi* S167T polymorphism (analogous to that of levamisole resistance polymorphism S168T in *H. contortus*) was detected in only one population (Farm 7) and was present at a low frequency of 1.1%. However, the resistance allele was observed in two ASVs that clustered closely together phylogenetically. Fifty-seven *acr-8* ASVs were observed, generated from sequencing reads with depths ranging from 4,621-5,742 (mean = 5,297) per population.

## 4 Discussion

The results of this study align with global trends of increasing anthelmintic resistance and demonstrate that resistance to the three main anthelmintic classes exists in several nematode species infecting Scottish dairy cattle. Controlling GIN infections primarily relies on metaphylactic and therapeutic treatment with anthelmintics. In the UK and Europe, resistance in parasitic nematodes of cattle is not yet considered widespread and reported resistance levels have generally been low to moderate (Rose Vineer et al., 2020). The FECRT remains the most important field test for assessing the susceptibility or resistance status of GIN populations (Kotze et al., 2020). However, the original WAAVP guidelines for the FECRT (Coles et al., 1992) had not been updated for over 30 years until Kaplan et al. (2023) recently revised them. These revised guidelines not only revise the recommendations for study design and reporting but also alter the criteria (cut-offs) for identifying resistant populations. Additionally, several statistical approaches have been proposed for analysing FECRT data and accounting for sources of variation (Denwood et al., 2023; Torgerson et al., 2014). It is important to recognise that applying different guidelines and statistical methods can lead to varying interpretations of the same FECRT results. Ehnert et al. (2025) conducted FECRTs on cattle farms in Germany and found that the level of agreement between two statistical models was consistent with the level of agreement in the current study, although there was low agreement between the original and revised guidelines. In the current study, it should be emphasised that many of the populations deemed resistant under the revised guidelines were classified as susceptible according to the original guidelines. This complicates the comparison of findings across studies using different guidelines, as the original guidelines are likely to underestimate the prevalence of resistant strongyle populations.

To achieve the necessary statistical power to determine species-specific resistance status, multiple FECs were required per sample to count a sufficient number of eggs, while only one FEC was sufficient to establish whether resistance was present within the population. Determining species-specific efficacy presents several statistical challenges, as even with pre- and post-treatment individual efficacy estimates, assumptions must be made about variation in relative species abundance among individuals. These assumptions include: that species proportions remain consistent across animals; that individuals with high total FECs do not systematically differ in species composition from those with low FECs; that individual efficacy reflects species-specific efficacy linearly; that variance in efficacy between animals is not driven by species heterogeneity; and that measurement errors are comparable across all species. The agreement between statistical models of the interpolated species resistance status was poor across both farms and treatments. The credible intervals generated by *bayescount* were consistently wider than those of *eggCounts,* even when point estimates are similar. This is typical of nonparametric models, and assumptions about species heterogeneity exacerbate CrI instability. This is most pronounced when CrIs are narrower; both methods tend to agree, but when CrIs are wider—as seen in the interpolated species data—small changes in interval width or threshold position can cause resistance status to change, when the drugs are still highly effective.

The nemabiome analysis revealed that *O. ostertagi* and *C. oncophora* were the most prevalent and abundant species in young cattle. This finding aligns with other UK and European studies that have demonstrated these species are the most common (Roeber et al., 2017; Baltrušis et al., 2019). The number of species identified in pre-treatment and organic populations ranged from four to eight, totalling 11 species across six genera. Nemabiome analysis currently represents the most detailed technique to characterise strongyle nematode communities. The large volume of data generated allows for the detection of rare species that are infrequently studied, but species identification requires prior knowledge of DNA sequences for each species and selection of the right species or genera should be done with care. In most research, read counts are used as a proxy for abundance, however PCR and sequencing bias, copy number variants and differing numbers of cells between species can all influence the relative read abundance (Avramenko et al., 2015).

After treatment with ML, all farm populations showed an increase in the proportion of *Cooperia* spp. The generally lower clinical pathogenicity of *Cooperia* spp. compared with other gastrointestinal nematodes, although still associated with reduced weight gain in young cattle, may partly explain why farmers seldom report obvious signs of resistance. Furthermore, the limited sensitivity of the FECRT, which requires at least 25% of a population to be resistant (Martin et al., 1989), combined with the higher fecundity of *Cooperia* spp., probably underestimates efficacy against the less fecund *O. ostertagi* (Hansen and Perry, 1994). In New Zealand, where many cases of GIN resistance to MLs have been well documented, there are still no reports of clinical parasitism caused by this species (Jackson et al., 2006). Additionally, the concentration of IVM in the abomasal mucosa that *O. ostertagi* encounters is higher than that of the intestine, the target site of *C. oncophora* (Lifschitz et al., 2000). This may be one reason why *Cooperia* spp. are the dose-limiting GIN and therefore were anticipated to be the first species to develop ML resistance in cattle (Coles, 2002).

In this study, the standardised egg hatch test protocol using thiabendazole was employed to compare *in vitro* assay data, BZ-treatment FECRT data, and BZ-resistance allele frequency data. It is widely accepted that the EC_50_ cut-off value for BZ-resistance is 0.1 μg TBZ/mL (Coles et al., 2006). The EC values reported in this study (EC_50_: 0.023-0.15 μg TBZ/mL, EC_95_: 0.08-1.07 μg TBZ/mL) were significantly higher than those obtained in a similar European study (von Samson-Himmelstjerna et al., 2009), where pre-treatment EC_50_ values of 0.027-0.038 μg TBZ/mL were observed for a mixed species population. However, only one publication has reported EC_50_ values for known susceptible *O. ostertagi* and *C. oncophora* populations, which ranged from 0.022-0.034 μg and 0.04-0.052 μg, respectively (Demeler et al., 2013). An EC_50_ value (0.108-0.118 μg) for a known-resistant *O. ostertagi* population (*O.o. Hamilton, 2010*) has been documented (Demeler et al., 2013), but no efficacy data from an FECRT or controlled efficacy test have been provided. The notably high EC_50_ and EC_95_ values for some populations undoubtedly suggest the presence of a resistant population or subpopulation.

The mixed amplicon sequencing approach successfully identified known BZ-resistance alleles, present in 0-43.8% of all *β-tubulin isotype-1* reads, and observed to increase significantly after FBZ treatment on Farm 1. The F200Y variant was the most commonly observed resistance allele in *O. ostertagi* and was the only resistance allele observed in *C. oncophora*. However, among *Trichostrongylus* spp., the E198L variant was the most prevalent in 5/6 populations and was only more abundant on Farm 14. The absence of a clear relationship between EC values and the frequency of *β-tubulin isotype-1* resistance alleles likely represents the influence of multiple biological and technical factors. Previous studies have demonstrated the clear correlation between the F200Y resistance allele frequency and EC_50_ values in *H. contortus* isolates (Baltrušis et al., 2020; Samson-Himmelstjerna et al., 2009), but no such correlation was seen in *C. oncophora*. However, these studies utilised laboratory isolates of a single species. The presence of multiple species within each field population likely masks the true EC values for each species and reduces the statistical power of each assay. Additionally, the inability of pooled amplicon sequencing to link genotype to individual phenotypes further obscures direct correlations. These results underscore that EC values and allele frequencies of field populations (at least when obtained by amplicon sequencing of pools) should be interpreted as complementary rather than interchangeable, indicators of resistance.

The S167T variant is analogous to that of the S168T, which has been shown to be highly correlated with levamisole resistance in *H. contortus* (Antonopoulos et al., 2024), and was present at a very low frequency in two *O. ostertagi* populations. The same analogous S167T variant in *T. circumcincta* has been observed at high frequencies in one levamisole-resistant isolate, which had been sequenced following multiple rounds of levamisole treatment, but not in two other levamisole resistant isolates, which had not been recently treated (McIntyre et al., 2025). The detection of S167T in *O. ostertagi* is a novel finding and has not been previously reported in this species but previously reported in field populations of *Haemonchus* spp. from cattle (Francis and Šlapeta, 2024). Although it was present at low frequency, this potential resistance allele was identified in two ASVs, making it unlikely to be a PCR artefact.

## 5 Conclusion

This study examined the anthelmintic efficacy of the main compounds used to treat cattle in the UK, using both *in vivo* and *in vitro* assays, and highlighted the variation in efficacy between farms and strongyle species. It demonstrates emerging resistance to MLs and BZs in cattle nematodes and emphasizes the need for harmonised diagnostic guidelines and sustainable control strategies. The variability in treatment efficacy is clear, as well as the influence of different statistical methods and diagnostic criteria on resistance classification. The revised FECRT guidelines complicate comparison with the previous studies when efficacy is ∼90% and highlight the importance of consistent methodology. The higher-than-expected BZ EC values for *O. ostertagi* and *C. oncophora* are of interest and warrant further investigation, given the limited empirical data and guidance on interpreting and assessing resistance using the EHT. The assessment of BZ efficacy on 48% of Scottish organic dairy farms represents a significant coverage of the national organic cattle sector, strengthening the representativeness of the findings. The variable frequency of BZ-resistance alleles, with high relative read abundance in some populations, is notable because if the trend of using BZ products against GIN infections in UK cattle continues, selection for resistance will likely increase. The high abundance of BZ resistance alleles on organic farms and higher than expected EC values is of great concern as these farms almost exclusively rely on this class and are restricted from using MLs. Therefore, ongoing and improved efficacy testing, to promote timely action, would be highly advantageous.

## CRediT authorship contribution statement

**Paul Campbell:** Conceptualization, Data curation, Formal analysis, Investigation, Methodology, Project administration, Writing – original draft, Writing – review & editing. **Jennifer Mcintyre:** Conceptualization, Methodology, Supervision, Visualization, Writing – review & editing. **Alistair Antonopoulos:** Conceptualization, Investigation. **Kerry O'Neill:** Investigation. **Andrew Forbes:** Conceptualization, Formal analysis, Methodology, Supervision, Writing – review & editing. **Kathryn Ellis:** Conceptualization, Formal analysis, Funding acquisition, Methodology, Supervision, Writing – review & editing. **Roz Laing:** Conceptualization, Formal analysis, Funding acquisition, Methodology, Resources, Supervision, Writing – review & editing.

## Declaration of competing interest

The authors declare that they have no known competing financial interests or personal relationships that could have appeared to influence the work reported in this paper.

## Data Availability

The data for this study have been deposited in the European Nucleotide Archive (ENA) at EMBL-EBI under accession number PRJEB105434 (https://www.ebi.ac.uk/ena/browser/view/PRJEB105434). Code used in this project is available at (https://github.com/pau1campbe11/Characterising-AR-against-BZs-MLs-in-GIN-of-cattle).
